# Identifying the active components of Baihe–Zhimu decoction that ameliorate depressive disease by an effective integrated strategy: a systemic pharmacokinetics study combined with classical depression model tests

**DOI:** 10.1186/s13020-019-0254-9

**Published:** 2019-09-24

**Authors:** Ming Zhong, Xiaoting Tian, Shuoji Chen, Mingcang Chen, Ziqiong Guo, Minna Zhang, Gongpu Zheng, Zhixiong Li, Zhangpeng Shi, Guanghui Wang, Honggang Gao, Fang Liu, Chenggang Huang

**Affiliations:** 1 College of Pharmacy, Jining Medical University, Rizhao, 276826 People’s Republic of China; 2 Shanghai Research Center for Modernization of Traditional Chinese Medicine, Shanghai Institute of Materia Medica, Chinese Academy of Science, Shanghai, 201203 People’s Republic of China

**Keywords:** Baihe–Zhimu decoction, Antidepressant, Timosaponin BII, Timosaponin BIII, Drug discovery

## Abstract

**Background:**

Modern pharmacological studies have demonstrated that Baihe–Zhimu decoction (BZD) has antidepressant effects. However, the complex composition and lack of clear evaluation standards for BZD make it less likely to be understood and accepted than evidence-based active natural compounds.

**Methods:**

In this study, an effective method for the identification of antidepressant components was demonstrated and applied to BZD. The first step was to evaluate the efficacy of BZD by the forced swimming test (FST) and the tail suspension test (TST), followed by successive quantitative analyses of the absorbed constituents at different stages, such as before hepatic disposition, liver distribution, after hepatic disposition and brain distribution after the oral administration of BZD. Finally, the compounds detected in the brain were confirmed by activity testing.

**Results:**

Our investigation observed that timosaponin BII and timosaponin BIII were accurately determined in the brain after oral administration of BZD, and they were further confirmed to reduce the immobility time in the FST and TST. As described above, timosaponin BII and timosaponin BIII were used to scientifically and reasonably explain the effective chemical basis of the effect of BZD on depression.

**Conclusions:**

This research affords an effective method to discover lead molecules for antidepressants from traditional Chinese medicine.

## Background

Many Chinese medicine formulae have been used for the treatment of diseases in China and are also regarded as alternative therapeutic agents in other Asian countries [1, 2]. However, the chemical constituents in Chinese medicine formulae are so complex and diverse that some of them may be effective and others may not throughout the course of therapy. Therefore, the identification of the main effective components from Chinese medicine formulae will benefit the discovery of lead compounds and the optimization of new drug development. At present, the conventional phytochemical approach remains the main method for the discovery of effective components or effective component groups. Although they exhibit various bioactivities in vivo, some components may display extremely low bioavailability [3, 4]. Thus, they cannot be regarded as the main effective components of Chinese medicine formulae, because efficacy is considered to rely on bioactive compounds with sufficient exposure in the plasma or target organs [5, 6]. Hence, it is necessary to develop a strategy for screening bioactive components with high exposure from Chinese medicine formulae.

Our group used an effective method combining classical tests for specific activity and pharmacokinetics studies based on high-performance liquid chromatography coupled to triple quadrupole mass spectrometry (HPLC-QQQ MS) to screen for the main effective compounds in traditional Chinese medicine (TCM) [7–9]. The first step was to evaluate the efficacy of crude extracts and perform quantitative analysis of their major chemical constituents. Then, the absorbed constituents were quantitatively analyzed in successive stages in vivo after the oral administration of crude extracts, such as before hepatic disposition (in the portal vein plasma), during liver distribution, after hepatic disposition (in the systemic plasma) and during target organ distribution. Finally, the compounds detected in the target organ were confirmed by activity testing and further mechanistic research.

Baihe–Zhimu decoction (BZD), consisting of two herbs, Baihe (*Lilium brownii var. viridulum*) and Zhimu (*Rhizoma anemarrhenae*), is a traditional prescription to treat lily disease, which exhibits similar symptoms to depression. Modern pharmacological studies have demonstrated that BZD has antidepressant effects in animal models [10, 11]. However, the complex composition of BZD and the lack of clear evaluation standards make its use less likely to be understood and accepted than that of evidence-based active natural compounds. Therefore, we have previously characterized a total of 39 compounds in the BZD to understand its chemical basis [12]. Among them, flavonoids and steroid saponins are considered the most important bioactive constituents [13–15]. Moreover, we found that the levels of mangiferin, neomangiferin, timosaponin BII, timosaponin BIII and timosaponin AIII in the portal vein plasma and systemic plasma were all above the detection limit of HPLC-QQQ MS [16], which could contribute to the in vivo process of BZD.

**Fig. 1:**
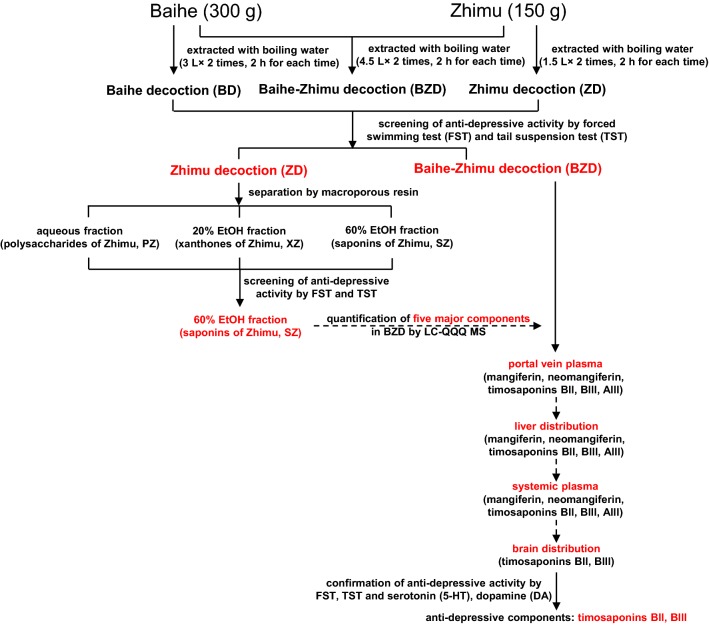
Procedure for the discovery of antidepressant components in BZD

In this study, we tried to discover the effective components of BZD for the treatment of depression using the strategy described above. After evaluation of the antidepressant activity of BZD, each individual herb and the corresponding fractions, the major components in BZD were quantified by HPLC-QQQ MS. Then, the absorbed constituents were determined in several stages after oral administration of BZD. The brain is the main target organ for mental disease, but little information related to brain disposition after oral administration of BZD in rats was available. Therefore, the pharmacokinetic behavior in the brain was finally tested, and the antidepressant activity of the detected components in the brain was validated. A flowchart of this study is shown in Fig. 1. This research provided an effective method for the discovery of the main effective components in Chinese medicine formulae.

## Methods

### Materials and reagents

Baihe (*Lilium brownii var. viridulum*) and Zhimu (*Rhizoma anemarrhenae*) were purchased from Shanghai Kangqiao Chinese Medicine Tablet Co., Ltd. (Shanghai China) and authenticated by Dr. Zhixiong Li (Shanghai Research Center for Modernization of TCM, Shanghai Institute of Materia Medica, Shanghai, China). Mangiferin, neomangiferin, timosaponin BII, timosaponin BIII and timosaponin AIII (purity > 98%) were supplied by Chengdu Biopurify Phytochemicals Co., Ltd. (Chengdu, China), and fluoxetine hydrochloride was provided by Eli Lilly & Co., Ltd. (Indianapolis, USA). HPLC-grade agents, including methanol, acetonitrile and formic acid, were obtained from Merck & Co., Inc. (Darmstadt, Germany). All other chemicals were purchased from Sinopharm Chemical Reagent Co., Ltd. (Shanghai, China) and were of analytical grade. The deionized water was purified by a Milli-Q system (Millipore, Billerica, MA, USA).

### Preparation of Baihe–Zhimu decoction, individual herbs and related fractions

Baihe (300 g) and Zhimu (150 g) were cut into slices and mixed together at a weight ratio of 2:1. Then, the mixture (450 g) was extracted twice with boiling water (4500 mL) for 2 h each time. After filtration, the supernatant was condensed to 300 mL under reduced pressure to obtain Baihe–Zhimu decoction (BZD). The chemical characteristics of BZD were further investigated by HPLC-QQQ MS to ensure the chemical consistency of the tested sample. Five major components, including timosaponin BII, timosaponin BIII, mangiferin, neomangiferin and timosaponin AIII, were identified by comparing the retention times with those of the chemical standards, and their concentrations were determined to be 8979.80, 4191.10, 2649.02, 1624.27 and 442.91 μg/g BZD, respectively. In addition, each herb in BZD was individually extracted with boiling water using the same method as motioned above. The prepared Zhimu decoction (ZD) was fractionated by microporous resin to obtain three fractions, including polysaccharides of Zhimu (PZ, elution with water), xanthones of Zhimu (XZ, elution with 20% EtOH) and saponins of Zhimu (SZ, elution with 60% EtOH).

### HPLC-QQQ MS conditions

The HPLC-QQQ MS method was developed according to a previous study in our group [16]. Briefly, five compounds were simultaneously determined in plasma, liver and brain samples using a 1260 Series liquid chromatography system (Agilent Technologies, Palo Alto, CA, USA) coupled to a 6460 triple quadrupole mass spectrometer with an electrospray ionization source (Agilent Technologies, Palo Alto, CA, USA). Chromatographic separation was performed on an ACE Super C18 column (100 mm × 2.1 mm, 3.0 µm, Advanced Chromatography Technologies Ltd. Aberdeen, Scotland) at a temperature of 40 °C. The mobile phase consisted of water containing 0.1% formic acid (A) and acetonitrile containing 0.1% formic acid (B). The gradient program was performed as follows: 0–3 min, 92% A; 3.5–4.0 min, 88–60% A; 5.5–6 min, 60–55% A; 6.6–7 min, 55–5% A; 11–11.01 min, 5–92% A; 11.01–13.5 min, 92% A. The flow rate was set to 0.35 mL/min in the time range of 0–6.6 min and kept at 0.45 mL/min between 7 and 13.5 min. Five components were monitored in negative multiple reaction monitoring (MRM) mode. The parameters of the mass spectrometer were as follows: capillary voltage, 3500 V; nebulizer, 45 psi; gas temperature, 350 °C; gas flow rate, 12 L/min; sheath gas temperature, 400 °C; sheath gas flow rate, 8 L/min. MassHunter Workstation software (Agilent Technologies, Palo Alto, CA, USA) was used for system operation and data analysis.

### Animals and treatments

All Sprague–Dawley (SD) rats and ICR mice were purchased from Shanghai SLAC Laboratory Animal Co., Ltd. and kept in the breeding room at 22 ± 2 °C and 50 ± 10% humidity in Shanghai Institute of Materia Medica (SIMM). The experimental protocols were approved by the Institutional Animal Care and Use Committee in SIMM. After 1 week of acclimation, all the SD rats were orally administered BZD (15 g/kg). The biological samples were collected from the rats after fasting for 12 h. After anaesthetization, the rats were dissected at 5 min, 15 min, 30 min, 1 h, 2 h, 4 h, 7 h, 10 h, 20 h and 40 h (*n* = 5 for each time-point). The hepatic portal vein plasma (2 mL) and the systemic plasma (6–8 mL) were subsequently harvested, and finally the liver and brain tissue samples were collected. The plasma sample were centrifuged at 9500×*g* for 5 min, and the tissue samples were washed with saline solution. All samples were stored at − 80 °C until analysis. The samples were prepared according to the method described in the previous study by our group [16].

The antidepressant activity of the drugs was evaluated by classic behavioral measurements, such as the forced swimming test (FST) and tail suspension test (TST). All the ICR mice were randomly divided into several groups (n = 8) as follows: a control group with stimulation and different test groups subjected to FST and TST after the oral administration of different drugs for 8 days, including fluoxetine (10 mg/kg), BZD (3 g/kg), ZD (3 g/kg), Baihe decoction (BD, 3 g/kg), PZ (21.2 mg/kg), XZ (21.2 mg/kg), SZ (21.2 mg/kg), timosaponin BII (10 mg/kg) and timosaponin BIII (10 mg/kg).

### Behavioral assessment

The FST was performed according to a previously described method with minor modifications [17]. Briefly, each mouse was individually placed in an open water-filled cylinder (H: 50 cm; Ø: 20 cm; water depth: 35 cm; temperature: 23–25 °C) and allowed to swim for a period of 6 min. The total time of immobility was recorded during the last 4 min of the testing duration. Immobility is defined as the mice floating in the water without movement. The TST was conducted as described in the literature [18]. Briefly, the mice were suspended 20 cm above the floor for 6 min. The time of immobility was recorded after the first 2 min. Mice in a completely motionless state were considered immobile.

### Determination of monoamine neurotransmitter levels

The contents of dopamine (DA) and serotonin (5-HT) in the plasma were determined using ELISA Kits (Shanghai Jianglai Biotech Co., Ltd., Shanghai, China) according to the manufacturer’s instructions.

### Data analysis

A noncompartmental analysis was carried out using WinNonlin software (Pharsight 6.2, NC, USA) to calculate the PK parameters. The significance of the results in behavior assessment was analyzed by unpaired Student’s *t*-tests. A P value less than 0.05 was considered significant. The liver extraction ratio (ER) indicated the fraction of hepatic clearance and first-pass effect, and the calculation formula was as reported in a previous study by our group [16].

## Results

The forced swimming test (FST) and the tail suspension test (TST) together were performed to evaluate the antidepressant effect of BZD. As described in Table 1, BZD and fluoxetine both markedly attenuated the immobility time in the FST (P < 0.05) and TST (P < 0.01) by comparison with that of control mice, suggesting that BZD could alleviate depressive disorder. To further evaluate the contribution of the individual herbs in BZD in relieving depressive symptoms, the extracts of each herb were similarly applied in FST and TST. The results showed that the Zhimu decoction (ZD) also caused a significant reduction in immobility time in both tests compared with that of control mice (P < 0.05), indicating that the individual herbal medicine Zhimu could ameliorate depressive-like behavior. Meanwhile, there was no significant decrease in the duration of immobility after treatment with Baihe decoction (BD). Thus, the individual herb Zhimu may play a major role in relieving depression. Based on the analysis of the components of BZD, the extract of Zhimu was further separated into different fractions by microporous resin, including the polysaccharides of Zhimu (PZ), xanthones of Zhimu (XZ) and saponins of Zhimu (SZ), for screening for antidepressant activity. As shown in Table 2, the immobility time of the SZ-treated group was significantly decreased compared with that of the control group. No significant differences were observed in the PZ-treated and XZ-treated groups. These results indicated that SZ should be considered the main active fraction.

**Table 1:** Effects of BZD, ZD and BD on the immobility time of ICR mice in FST and TST

Groups	Dosage	Immobility time (mean ± SD, s)	
		FST	TST
Control	–	127.6 ± 40.9	109.1 ± 15.5
Fluoxetine	10 mg/kg	84.8 ± 15.2*	62.0 ± 36.2**
BZD	3 g/kg	90.1 ± 26.4*	67.3 ± 35.8**
ZD	3 g/kg	85.9 ± 28.2*	83.0 ± 23.0*
BD	3 g/kg	92.4 ± 38.2	95.6 ± 15.9

BZD, ZD and BD represented Baihe–Zhimu decoction, Zhimu decoction and Baihe decoction, respectively

FST and TST represented forced swimming test and tail suspension test, respectively

* P < 0.05 and ** P < 0.01 compared with control group

**Table 2:** Effects of different fractions of Zhimu on the immobility time of ICR mice in FST and TST

Groups	Dosage (mg/kg)	Immobility time (mean ± SD, s)	
		FST	TST
Control	–	134.3 ± 22.1	118.6 ± 18.1
Fluoxetine	10	82.0 ± 21.3**	69.5 ± 21.5*
SZ	21.2	86.8 ± 18.2**	70.0 ± 21.1*
PZ	21.2	104.6 ± 27.7	102.6 ± 24.5
XZ	21.2	119.3 ± 19.9	105.3 ± 26.3

SZ, PZ and XZ represented saponins of Zhimu, polysaccharides of Zhimu and xanthones of Zhimu, respectively

FST and TST represented forced swimming test and tail suspension test, respectively

* P < 0.05 and ** P < 0.01 compared with control group

### Pharmacokinetic study of five major components after oral administration of BZD

The HPLC-QQQ MS method has already been developed according to the previous study in our group [16]. In brief, full validation of the selectivity, linearity, accuracy, precision, matrix effect, extraction recovery, and stability, was performed for the simultaneous determination of two xanthones (neomangiferin and mangiferin) and three saponins (timosaponin BII, timosaponin BIII and timosaponin AIII) in the biological matrix by HPLC-QQQ MS. These results confirmed that two xanthones and three saponins in the biological matrix could be simultaneously determined by developed method [16]. Based on the developed method, five major components, including timosaponin BII, timosaponin BIII, mangiferin, neomangiferin and timosaponin AIII, were selected for simultaneous determination and pharmacokinetic analysis in the portal vein plasma, liver tissue, systemic plasma and brain tissue. The related pharmacokinetic parameters are summarized in Table 3.

**Table 3:** The PK parameters of five major components of Zhimu in the portal vein plasma, liver, systemic plasma and brain (containing hippocampus and cerebellum) after oral administration of BZD at 15 g/kg in rats (n = 5)

PK parameters	Mangiferin		Neomangiferin		Timosaponin AIII	
	Portal vein plasma	Systemic plasma	Portal vein plasma	Systemic plasma	Portal vein plasma	Systemic plasma
T_1/2_ (h)	4.08	7.70	0.93	1.33	10.33	11.16
T_max_ (h)	1.00	1.00	0.25	0.25	4.00	4.00
C_max_ (ng/mL or ng/g)	1757.12 ± 219.18	889.36 ± 191.49	64.64 ± 12.00	125.28 ± 15.12	61.79 ± 25.59	16.04 ± 5.16
AUC_0–t_ (ng/mL or ng/g)	4020.89 ± 397.02	4217.27 ± 177.38	34.36 ± 2.60	78.15 ± 5.66	582.66 ± 104.67	156.12 ± 21.63
ER	− 4.88%		− 127.44%		73.20%	

PK parameters	Timosaponin BII		Timosaponin BIII			
	Portal vein plasma	Systemic plasma	Portal vein plasma	Systemic plasma		
T_1/2_ (h)	118.97	35.78	16.59	22.82		
T_max_ (h)	0.25	0.25	0.25	0.25		
C_max_ (ng/mL or ng/g)	880.38 ± 159.95	276.76 ± 48.41	226.39 ± 43.92	114.14 ± 13.38		
AUC_0–t_ (ng/mL or ng/g)	1094.92 ± 183.89	975.80 ± 253.95	369.20 ± 74.63	326.47 ± 54.29		
ER	10.88%		11.57%			

PK parameters	Mangiferin	Neomangiferin	Timosaponin AIII	Timosaponin BII	Timosaponin BIII	
	Liver	Liver	Liver	Liver	Liver	
T_1/2_ (h)	59.13	13.47	12.18	116.03	13.81	
T_max_ (h)	2.00	10.00	20.00	0.08	0.08	
C_max_ (ng/mL or ng/g)	130.59 ± 9.76	43.15 ± 2.51	9361.26 ± 243.85	301.69 ± 102.09	158.66 ± 22.12	
AUC_0–t_ (ng/mL or ng/g)	2520.63 ± 118.68	751.52 ± 87.96	199,598.41 ± 7787.31	6518.05 ± 411.22	859.42 ± 120.72	

PK parameters	Timosaponin B II	Timosaponin BII	Timosaponin BIII	Timosaponin BIII		
	Hippocampus	Cerebellum	Hippocampus	Cerebellum		
T_1/2_ (h)	27.41	16.17	1.52	20.65		
T_max_ (h)	10.00	0.25	1.00	0.25		
C_max_ (ng/mL or ng/g)	371.90 ± 153.80	1168.66 ± 93.39	198.23 ± 122.31	349.41 ± 34.90		
AUC_0–t_ (ng/mL or ng/g)	9093.43 ± 1034.21	11,816.11 ± 1035.46	2559.67 ± 928.52	3422.98 ± 373.46		

### Pharmacokinetic study before hepatic disposition

**Fig. 2:**
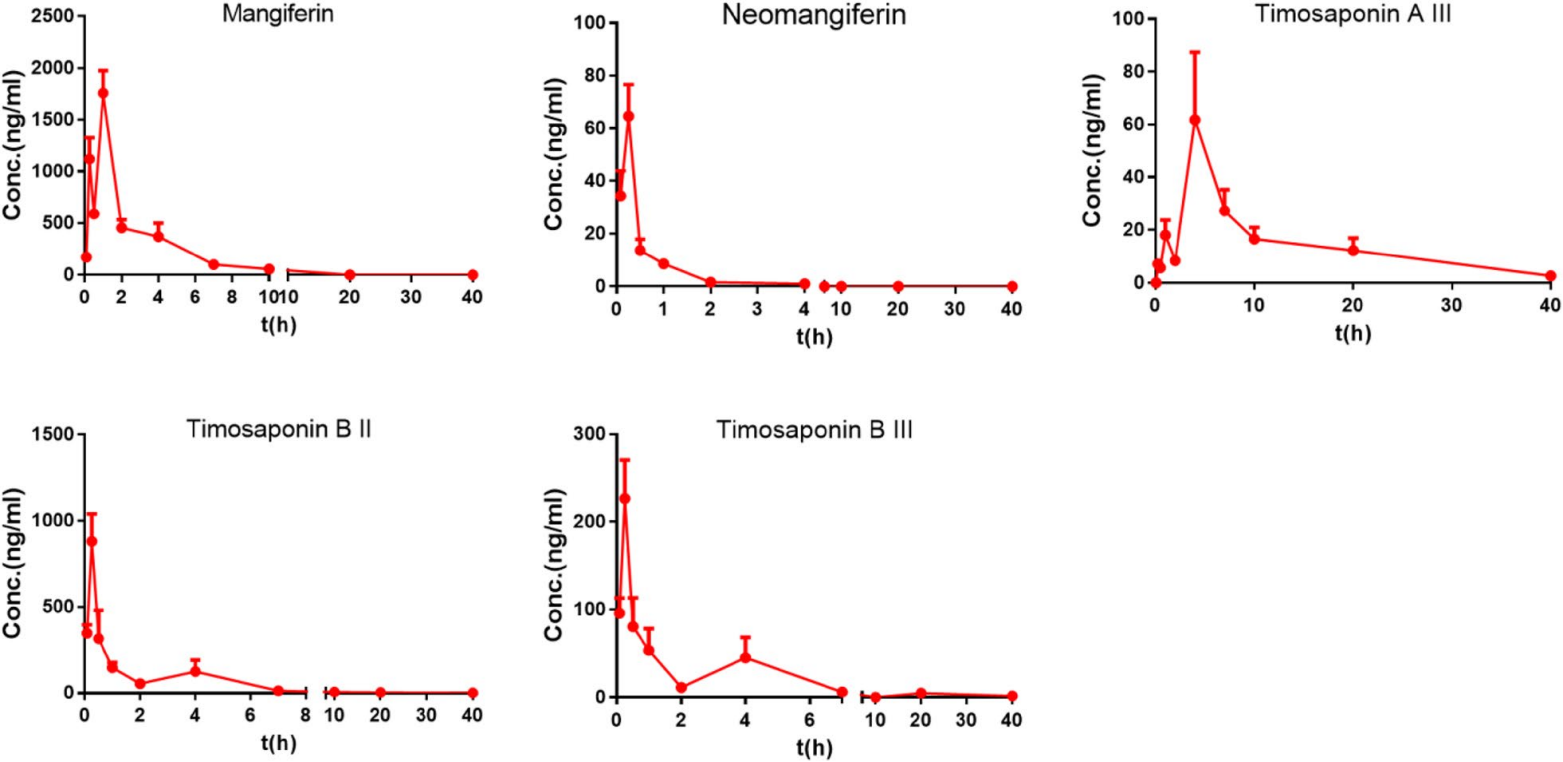
Mean concentration–time curves of the major chemical constituents in the portal vein plasma after the oral administration of BZD at 15 g/kg in rats (n = 5)

Portal vein plasma is the site after gut absorption but before hepatic disposition. As displayed in Fig. 2, five compounds were accurately quantified in the portal vein plasma after oral administration of BZD. Figure 2 shows that timosaponin BII, timosaponin BIII, mangiferin and timosaponin AIII exhibited the double-peak phenomenon in the concentration–time curves, which may be caused by enterohepatic recirculation. The plasma concentrations of timosaponin BII, timosaponin BIII, mangiferin, neomangiferin and timosaponin AIII reached a maximum plasma concentration (C_max_) at 880.38 ± 159.95 ng/mL, 226.39 ± 43.92 ng/mL, 1757.12 ± 219.18 ng/mL, 64.64 ± 12.00 ng/mL and 61.79 ± 25.59 ng/mL, respectively. The T_max_ values of timosaponin BII, timosaponin BIII and neomangiferin were greater than those of mangiferin and timosaponin AIII, and the rank order of t_1/2_ was timosaponin BII > timosaponin BIII > timosaponin AIII > mangiferin > neomangiferin. This result revealed that timosaponin BII was eliminated more slowly. The area under the concentration–time curve (AUC) is usually regarded as the objective marker for exposure to chemical components and as predictive of pharmacological responses [19]. The AUC values of timosaponin BII, timosaponin BIII, mangiferin, neomangiferin and timosaponin AIII were 1094.92 ± 183.89 ng/mL, 369.20 ± 74.63 ng/mL, 4020.89 ± 397.02 ng/mL, 34.36 ± 2.60 ng/mL and 582.66 ± 104.67 ng/mL, respectively. The large AUC values of the four compounds other than neomangiferin indicated good absorption and utility in the portal vein plasma.

### Liver distribution

**Fig. 3:**
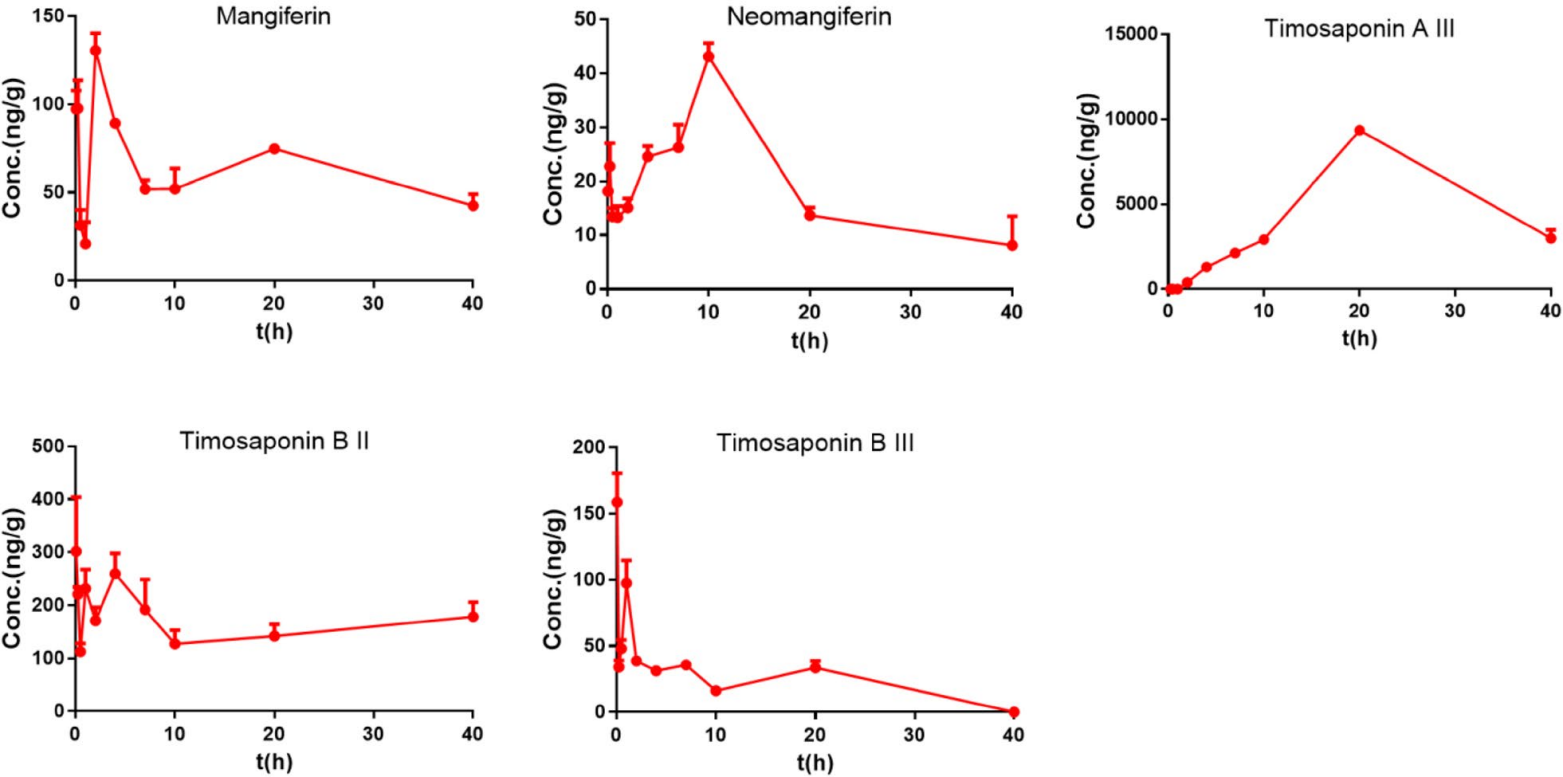
Mean concentration–time curves of the major chemical constituents in the liver tissue after the oral administration of BZD at 15 g/kg in rats (n = 5)

As displayed in Fig. 3, five compounds were determined accurately in the liver after oral administration of BZD. In the liver, timosaponin AIII had the maximum exposure, and the AUCs of timosaponin BII, timosaponin BIII, mangiferin, neomangiferin and timosaponin AIII were 6518.05 ± 411.22 ng/g, 859.42 ± 120.72 ng/g, 2520.63 ± 118.68 ng/g, 751.52 ± 87.96 ng/g and 199,598.41 ± 7787.31 ng/g, respectively. These values were different from those in the portal vein plasma, especially the highest value of timosaponin AIII. These results suggested that timosaponin AIII largely accumulated in the liver.

### Pharmacokinetic study after hepatic disposition

**Fig. 4:**
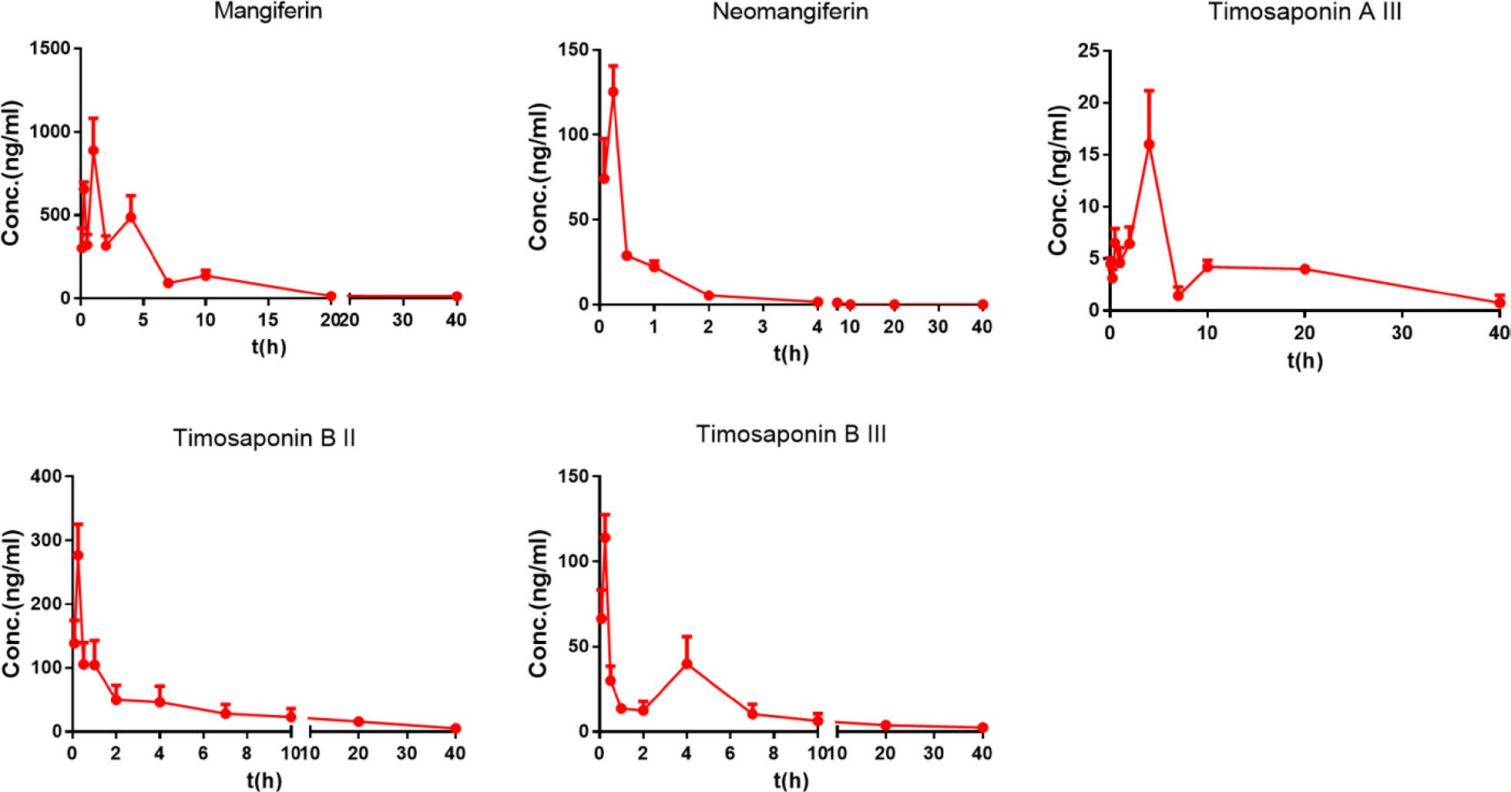
Mean concentration–time curves of the major chemical constituents in the systemic plasma after the oral administration of BZD at 15 g/kg in rats (n = 5)

After hepatic disposition, timosaponin BII, timosaponin BIII, mangiferin, neomangiferin and timosaponin AIII were transported to the systemic plasma. Figure 4 shows the similar pharmacokinetic properties of the five compounds detected in the systemic plasma and the portal vein plasma. Mangiferin showed the maximum AUC, followed by timosaponin BII, timosaponin BIII, timosaponin AIII and neomangiferin, with values of 4217.27 ± 177.38 ng/mL, 975.80 ± 253.95 ng/mL, 326.47 ± 54.29 ng/mL, 156.12 ± 21.63 ng/mL and 78.15 ± 5.66 ng/mL, respectively. Except for mangiferin and neomangiferin, the AUCs of the other compounds were much higher in the portal vein plasma than in the systemic plasma, corresponding to the effective recovery (ER) of timosaponin BII, timosaponin BIII and timosaponin AIII at 10.88%, 11.57% and 73.21%, respectively. In contrast, the ER values of mangiferin and neomangiferin were − 4.88% and − 127.44%, respectively. These results indicated that some other components may be converted to mangiferin and neomangiferin after hepatic metabolism in vivo.

### Brain distribution

**Fig. 5:**
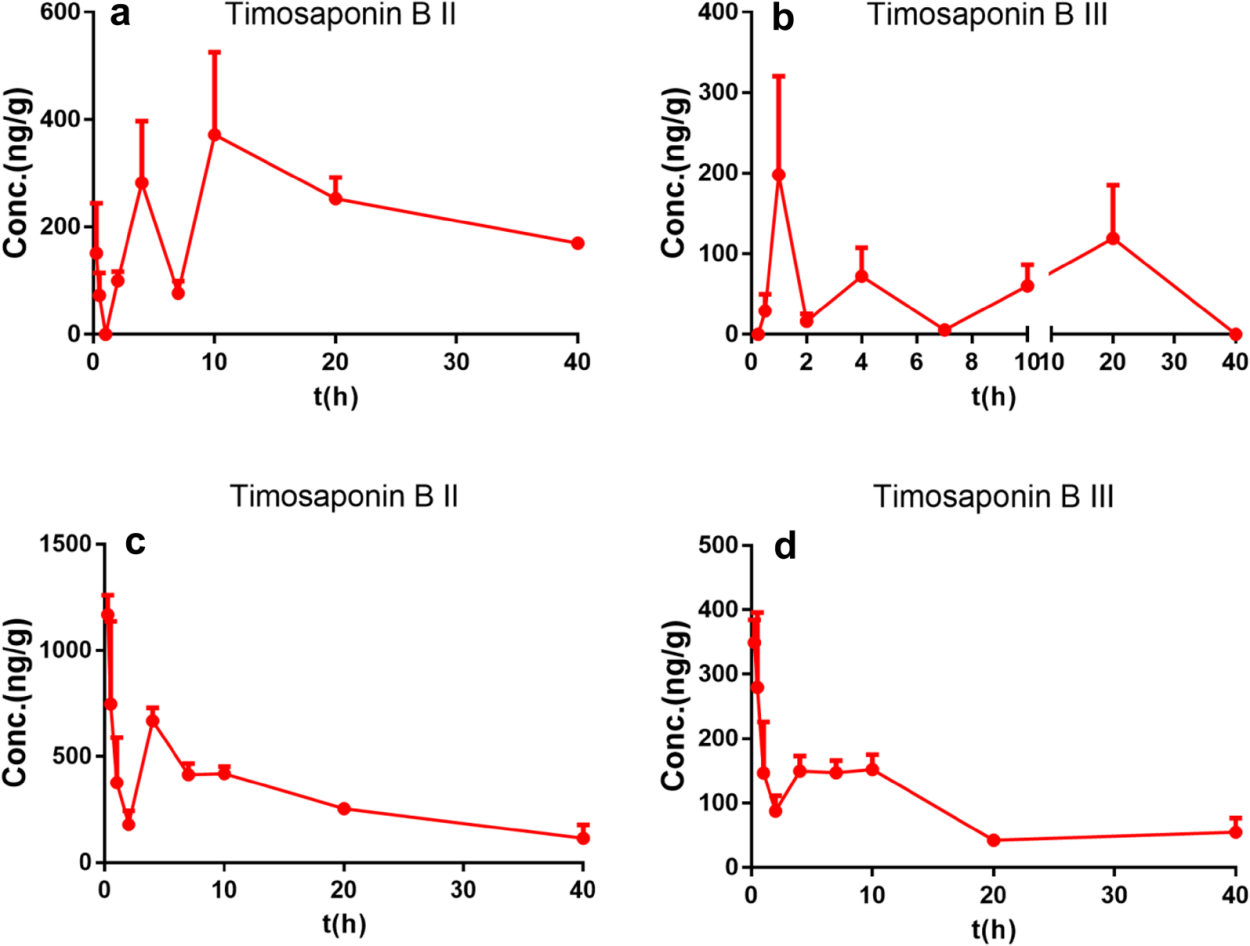
Mean concentration–time curves of timosaponin BII (**a**) and timosaponin BIII (**b**) in the hippocampus tissue and of timosaponin BII (**c**) and timosaponin BIII (**d**) in the cerebellum tissue after the oral administration of BZD at 15 g/kg in rats (n = 5)

As shown in Fig. 5, only two components were accurately quantified in the cerebellum and hippocampus after oral administration of BZD. Similar to those in the systemic blood, timosaponin BII and timosaponin BIII exhibited an obvious double-peak phenomenon in the time-concentration curves in the cerebellum. However, a multiple-peak phenomenon occurred in the hippocampus. This observation may be attributed to the multiple sites of intestinal absorption. The C_max_ and AUC of timosaponin BII were 371.90 ± 153.80 ng/g and 9093.43 ± 1034.21 ng/g, respectively, with a larger T_max_ at 10 h than that of timosaponin BIII (1 h). The C_max_ and AUC of timosaponin BIII were 198.23 ± 122.31 ng/g and 2559.67 ± 928.52 ng/g, respectively. These results indicated that timosaponin BII and timosaponin BIII, which achieved exposure in the brain tissue, may be the main effective components of BZD.

### Antidepressant activity of chemical components detected in brain

To verify the screening results of the method, the FST and the TST were employed to assess the antidepressant effects of the chemical compounds detected in the brain. As shown in Table 4, both timosaponin BII and BIII markedly attenuated the time of immobility compared with that of control mice in both the FST (P < 0.05) and TST (P < 0.01), suggesting that they could alleviate depressive disorder. Furthermore, the timosaponins BII and BIII were used to screen for the active mechanism by using 5-HT and DA assays. As shown in Table 5, the levels of 5-HT in the timosaponin BII-treated group and the timosaponin BIII-treated group were both significantly decreased compared with that of the control group. These results further confirmed that timosaponins BII and BIII should be considered effective components of BZD.

**Table 4:** Effects of timosaponin BII and timosaponin BIII on the immobility time of ICR mice in FST and TST

Groups	Dosage (mg/kg)	Immobility time (mean ± SD, s)	
		FST	TST
Control	–	118.1 ± 24.3	102.0 ± 16.9
Fluoxetine	10	70.2 ± 21.2^*^	50.0 ± 22.2*
Timosaponin B-II	10	72.1 ± 19.6^*^	48.2 ± 23.1*
Timosaponin B-III	10	68.6 ± 23.2^*^	58.4 ± 15.6*

FST and TST represented forced swimming test and tail suspension test, respectively

* P < 0.05 compared with control group

**Table 5:** Effects of timosaponin BII and timosaponin BIII on the content of 5-HT and DA

Groups	Dosage (mg/kg)	Content (ng/g)	
		5-HT	DA
Control	–	636.2 ± 80.1	171.6 ± 54.5
Fluoxetine	10	731.3 ± 104.6*	215.9 ± 36.5*
Timosaponin BII	10	716.5 ± 90.4*	196.9 ± 56.7
Timosaponin BIII	10	705.6 ± 47.7*	191.3 ± 69.8

5-HT and DA represented serotonin and dopamine, respectively

* P < 0.05 compared with blank group

## Discussion

Depression is a common psychiatric disorder that affects mental and physical health and involves a number of symptoms, including low mood, lack of happiness and attention, sleep disorders and fatigue, and feelings of guilt [20–22]. Although many synthetic antidepressant drugs have been used to treat depression, the therapeutic effects are unsatisfactory due to numerous side effects, such as insomnia, headache, and anxiety [23–25]. Therefore, it is urgent to identify promising alternative agents with greater efficacy and fewer undesirable effects.

In China, many Chinese medicine formulae, which are also regarded as alternative therapeutic agents in other Asian countries, have been used for the treatment of depression [26, 27]. Baihe–Zhimu decoction (BZD) is a classical prescription used to treat depression. However, the complex composition of BZD and lack of clear evaluation standards make it less well understood and accepted than better-studied natural active compounds. There are multiple ingredients in Chinese medicine formulae, but sufficient absorption into the plasma or target organs is required for them to be considered effective compounds. Therefore, as shown in Fig. 1, we developed an integrated method combining classical tests for specific activity and pharmacokinetic studies based on HPLC-QQQ MS to screen for BZD components that were effective against depression.

The forced swimming test (FST) and the tail suspension test (TST) are commonly used to investigate antidepressant activity. Our data showed that BZD and Zhimu decoction (ZD, aqueous extract of Zhimu) elicited a significant reduction in the immobility time in both tests, indicating that the single herb Zhimu could obviously improve depressive-like behaviors, whereas the single herb Baihe could not. Thus, the single herb Zhimu may play a more important role in relieving depression. The effective chemical base is regarded as a bioactive compound with adequate exposure in the plasma and liver tissue. Therefore, as described above, five major components from Zhimu, namely, timosaponin BII, timosaponin BIII, mangiferin, neomangiferin and timosaponin AIII, were selected for further study according to their bioactivities and higher content in the plasma and liver tissue [15, 16]. These compounds were then selected for quantitative analysis and pharmacokinetic study to evaluate their distribution in the brain after oral administration of BZD. Finally, two components detected in the brain, timosaponin BII and timosaponin BIII, were further validated by FST and TST to scientifically and reasonably explain the chemical basis of the effect of BZD on depression.

The results showed that timosaponin BII and timosaponin BIII clearly improved depressive-like behaviors. Moreover, Lu et al. confirmed the significant antidepressant effect of timosaponin BII in depressive rats, which was likely related to the content of 5-HT in the brain [28]. Zhang et al. had already demonstrated that timosaponin BIII significantly ameliorated depressive effects in rats by regulating inflammatory cytokines, BNDF signaling and synaptic plasticity [29]. We found that YY-21 and YY-23, two modified derivatives of timosaponin BIII, also exhibited antidepressant activity in the animal model [30, 31]. The validated results regarding the antidepressant activities of timosaponin BII and timosaponin BIII were consistent with previous literature reports. Thus, given their high brain exposure and obvious antidepressant effects in vivo, timosaponin BII and timosaponin BIII are probably predominantly responsible for the antidepressant effect of orally administered BZD.

## Conclusions

This investigation found that BZD, the individual herb Zhimu, and the saponins of Zhimu clearly ameliorated depressive-like behaviors evaluated by the forced swimming test (FST) and the tail suspension test (TST), whereas the individual herb Baihe did not, indicating that Zhimu may play a more important role in relieving depression. Based on the bioactivities and level of exposure in the plasma and liver tissue, five components, timosaponin BII, timosaponin BIII, mangiferin, neomangiferin and timosaponin AIII, were selected for quantitative analysis and pharmacokinetic characterization. Timosaponin BII and timosaponin BIII were accurately quantified in the brain after oral administration of BZD, and they were confirmed to improve depressive-like behaviors in the FST and TST. The levels of serotonin (5-HT) were also decreased after oral administration of timosaponin BII and timosaponin BIII. As described above, timosaponin BII and timosaponin BIII provided a rational explanation of the chemical basis for the effect of BZD on depression. The results of this study provide an effective method to discover lead compounds for depression therapy in traditional Chinese medicine.

## Data Availability

The datasets used in the current study are available from the corresponding author on reasonable request.

## Abbreviations

FST: forced swimming test
TST: tail suspension test
DA: dopamine
5-HT: serotonin
HPLC-QQQ MS: high-performance liquid chromatography coupled to triple quadrupole mass spectrometry
PK: pharmacokinetics
ER: extraction ratio
BZD: Baihe–Zhimu decoction
ZD: Zhimu decoction
BD: Baihe decoction
PZ: polysaccharides of Zhimu
XZ: xanthones of Zhimu
SZ: saponins of Zhimu
