# Correction to: Identifying the active components of Baihe–Zhimu decoction that ameliorate depressive disease by an effective integrated strategy: a systemic pharmacokinetics study combined with classical depression model tests

**DOI:** 10.1186/s13020-020-00312-2

**Published:** 2020-04-02

**Authors:** Ming Zhong, Xiaoting Tian, Shuoji Chen, Mingcang Chen, Ziqiong Guo, Minna Zhang, Gongpu Zheng, Zhixiong Li, Zhangpeng Shi, Guanghui Wang, Honggang Gao, Fang Liu, Chenggang Huang

**Affiliations:** 1 College of Pharmacy, Jining Medical University, Rizhao, 276826 People’s Republic of China; 2 Shanghai Research Center for Modernization of Traditional Chinese Medicine, Shanghai Institute of Materia Medica, Chinese Academy of Sciences, Shanghai, 201203 People’s Republic of China; 3 University of Chinese Academy of Sciences, Beijing, 100049 People’s Republic of China

## Correction to: Chin Med (2019) 14:37 10.1186/s13020-019-0254-9

In the original publication of this article [1], another affiliation (Affiliation 3: University of Chinese Academy of Sciences, Beijing 100049, People’s Republic of China.) for the author Ziqiong Guo is missing due to the carelessness during the author proof. In addition, in Affiliation 2 “Chinese Academy of Science” should be changed to “Chinese Academy of Sciences”.

The full author affiliation for Ziqiong Guo is Ziqiong Guo^2,3^:

^2^Shanghai Research Center for Modernization of Traditional Chinese Medicine, Shanghai Institute of Materia Medica, Chinese Academy of Sciences, Shanghai 201203, People’s Republic of China.

^3^University of Chinese Academy of Sciences, Beijing 100049, People’s Republic of China.

The authors sincerely apologize for the inconvenience caused to the readers.
